# Electroencephalography-Based Brain–Machine Interfaces in Older Adults: A Literature Review

**DOI:** 10.3390/bioengineering10040395

**Published:** 2023-03-23

**Authors:** Luca Mesin, Giuseppina Elena Cipriani, Martina Amanzio

**Affiliations:** 1 Mathematical Biology and Physiology, Department Electronics and Telecommunications, Politecnico di Torino, 10129 Turin, Italy; 2 Department of Psychology, Universitá di Torino, 10124 Turin, Italy

**Keywords:** brain–machine interfaces, brain–computer interfaces, electroencephalography, older adults

## Abstract

The aging process is a multifaceted phenomenon that affects cognitive-affective and physical functioning as well as interactions with the environment. Although subjective cognitive decline may be part of normal aging, negative changes objectified as cognitive impairment are present in neurocognitive disorders and functional abilities are most impaired in patients with dementia. Electroencephalography-based brain–machine interfaces (BMI) are being used to assist older people in their daily activities and to improve their quality of life with neuro-rehabilitative applications. This paper provides an overview of BMI used to assist older adults. Both technical issues (detection of signals, extraction of features, classification) and application-related aspects with respect to the users’ needs are considered.

## 1. Introduction

The aging of the world’s population is a major health challenge. This is the reason why new technologies based on human–machine interaction (HMI) or brain–machine interface (BMI) offer important perspectives to support older adults in their daily activities and improve their quality of life. As a heterogeneous process, aging refers to people of 60 years or older and needs to be studied in a comprehensive and multidimensional perspective that simultaneously considers different components and their interactions, i.e., a person’s *intrinsic capacity*, *environments,* and *functional ability*. Intrinsic capacity includes physical and mental functioning such as vitality and locomotor, sensory, cognitive, and psychological abilities; environment includes home, community, and society; functional ability integrates intrinsic capacity and how people interact with their environments. These three components and their domains can help personalize and prioritize care and services to meet the needs of older people from a comprehensive and person-centered perspective. In this direction, and according to the World Health Organization [1], addressing these components would enable the achievement of the Decade of Healthy aging 2021–2030. This under-researched topic could shed light on the process of describing and improving intrinsic capacities, the way people interact with their environment and functional abilities. This would be important for developing person-centered intervention programs to promote well-being and support patients with physical disabilities and dementia.

With this aim, many engineering tools, such as neurofeedback (NF) and brain–computer interface (BCI) technologies, have been developed to allow HMI that may be helpful in detecting possible age changes in healthy individuals and in rehabilitating patients with physical and cognitive impairments. In particular, recent reviews have emphasized the role of NF [2] and BCI [3] as countermeasures for the cognitive decline experienced during aging. For example, older adults with dementia may be assisted in their daily living activities [4].

The aim of this review on older adults is to examine BCI technology based on surface electroencephalogram (EEG), focusing on the intrinsic capacity to detect possible age changes and potential effectiveness of cognitive training (CT). We also provide an introductory tutorial on EEG processing and classification (in the Appendix A), which serve as fundamental tools for the development of HMI interfaces. Studies addressing these issues are proving critical to improving the functional abilities and well-being of older people, as outlined in the World Health Organization’s Guidelines for Healthy Aging [1].

## 2. Research Methodology

A systematic and comprehensive review of the literature on HMI to support aging has been applied to find, assess, and interpret significant research outcomes. The following steps of our research are detailed below: research strategy, inclusion/exclusion criteria, paper selection, and outcomes.

### 2.1. Search Strategy

To identify studies on HMI applied to assist older adults in their daily life activities and to improve their quality of life with diagnostic and neuro-rehabilitative applications, a systematic search strategy was implemented in the international literature online database PubMed. We entered the following query terms: (*Brain machine interfaces)* AND (*Ageing*), searching for relevant scientific literature published up to 27 October 2022.

**Figure 1:**
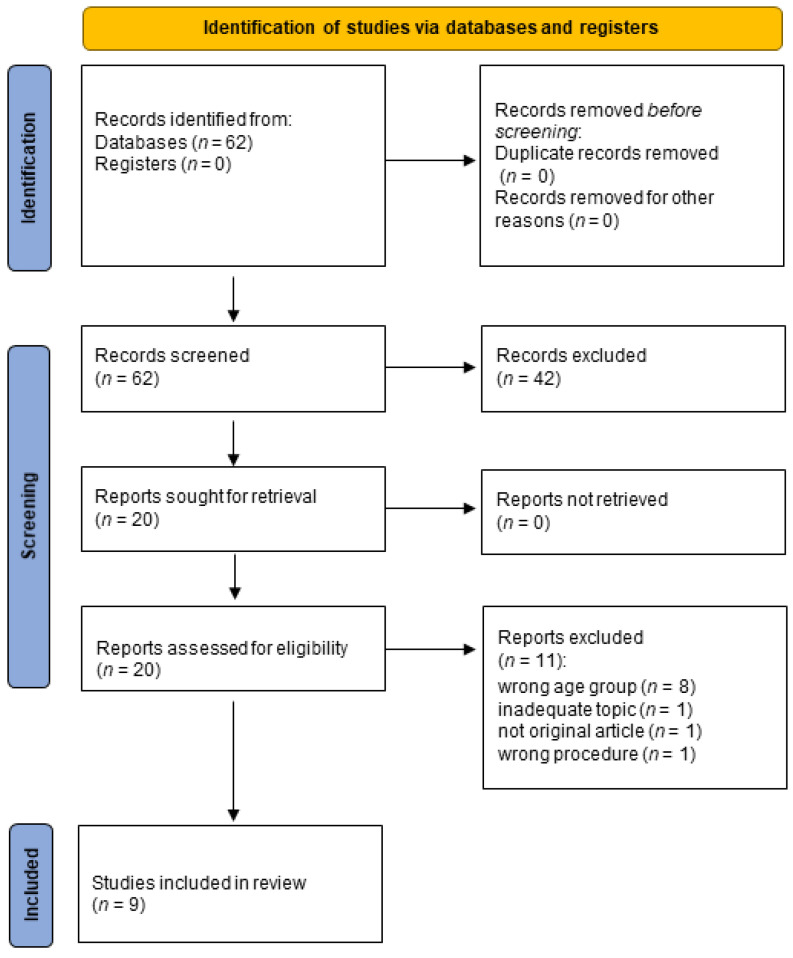
Study selection, following the PRISMA 2020 statement [5].

The results of both search queries are presented in the flowchart diagram shown in Figure 1. The guidelines of the Preferred Reporting Items for Systematic Review and Meta-Analyses (PRISMA) [5] were followed, adapted to our investigation.

Two authors (M.A. and G.E.C.) carried out the study selection process independently. They identified articles by title, abstract, and full text. Any disagreements were discussed and resolved. Finally, one author (L.M.) supervised this phase.

### 2.2. Inclusion and Exclusion Criteria

To guarantee the selection of pertinent articles, we included only studies satisfying the following criteria:

(a) HMI in older adults;
(b) HMI using EEG signals;
(c) Both healthy aging individuals and subjects with neurocognitive impairment;
(d) Both technical issues (signals detection, feature extraction, classification) and applicative implications (users’ need, effectiveness in restoration of lost skills, and rehabilitation).

The following exclusion criteria were adopted:

(a) Studies not using EEG signals;
(b) Subjects not meeting the ”ageing” criteria (mean age: 60 years and older) [6];
(c) Cross-sectional studies without differences between age groups (single-group data analysis).

### 2.3. Study Selection

After discussing the eligibility criteria for each study, two authors (M.A. and G.E.C.) independently analyzed the following information: population and its characteristics, sample size, procedures, study design, and outcomes. In case of disagreement, a consensus was reached or the judgment of a third author was sought (L.M.).

### 2.4. Outcome Measures

This review was conducted to find information about HMI in healthy aging subjects and in patients with neurocognitive disorders. The following information was also examined in relation to HMI: (a) technical issues; (b) applicative implications.

## 3. Results

### 3.1. Study Selection

The initial literature search process led to 62 articles meeting our eligibility criteria. Of these, none was removed before screening, as duplicates. After the identification and screening phase, 9 studies [7,8,9,10,11,12,13,14,15] were included and 53 were excluded (see flowchart, Figure 1). In particular, two studies on mild cognitive impairment were excluded, as wrong procedure (magnetic resonance imaging study) [16] and wrong age group [17]. The number and reasons for the exclusion of the articles were as follows:

- 23 did not take into consideration older adults (wrong age group);
- 19 were not original articles (12 reviews, 2 editorials, 2 conference papers, 1 perspective, 1 letter, 1 case);
- 6 focused on issues not concerning the aims of our review (inadequate topics);
- 4 studies did not evaluate humans (wrong species);
- 1 did not use EEG signals (wrong procedure).

All studies not included in this review and reasons for their exclusion are available in Table S1 in Supplementary Material.

### 3.2. Description of the Selected Studies

The included studies in healthy older adults focusing on HMI using EEG signals are described in the following sections. Different features were used to characterize the EEG and different methods were employed to translate the recorded information. The selected references are also summarized in Table 1, indicating main properties of the included subjects, and Table 2, regarding experimental protocol and processing. The processing methods are based on filtering and classification methods introduced in Appendix A, possibly with some modification (the reader is invited to refer to the references for details). Selected studies focus on specific intrinsic abilities and BCI assessment, such as: Cognition (e.g., memory, attention and motor imagery as a dynamic cognitive process in which a movement is mentally simulated without actually being performed), vitality (e.g., a reduction in energy in terms of fatigue), locomotor (physical movement), and sensory domains (such as vibro-tactile stimulation and sensitivity).

A further summary of the results and perspectives of the selected papers is shown in Table S2 in Supplementary Materials.

#### 3.2.1. Li et al., 2022 [7]

EEG characteristics are investigated during motor imagery (MI) by comparing young and older adults. A total of 20 healthy individuals participated in the study, including 10 young and 10 older adults. The authors analyzed both cognition and vitality by using BCI to examine the differences in terms of fatigue level, in both groups using left and right MI experiments. Fatigue is analyzed in terms of (1) discrimination of fatigue-sensitive channels in the parietal area, (2) calculation of rhythm entropy (RE) in the frontal area, and (3) assessment of synchronization of fatigue in the parietal lobe and EEG complexity in the frontal area. Data are analyzed using TFR and quantified by event-related desynchronization (ERD). Alpha, beta, and theta rhythms are extracted and used to quantify fatigue. Moreover, the cognitive activity is quantified by RE. The PLV between parietal and frontal lobes was also calculated. For the older population, a CNN is finally used for classification. The results show that older adults are less affected by the degree of cognitive fatigue during MI, compared to young participants. Nevertheless, MI energy is lower in the older population, than in younger people.

#### 3.2.2. Goelz et al., 2021 [8]

Possible age changes and differences in EEG data classification are investigated during active visuo-motor tasks. A total of 26 healthy individuals participated in the study, including 13 younger adults and 13 older adults. In this visuo-motor force control task, while seated in front of a computer screen, participants had to follow a target with their hand for five seconds by applying the required force. Force tracking was studied considering two task characteristics (sinusoidal and constant) with the right or left hand. A dimensionality reduction method called dynamic mode decomposition was used to extract brain network patterns. The tasks were classified using LDA. While comparing performance and patterns of brain activity between groups, authors found evidence of altered motor network function, in older subjects, suggesting de-differentiated and compensatory brain activation. These changes indicated less segregated activation of the motor brain network, even though they showed higher classification performance on the task feature. The results confirm an age-related reorganization of brain networks and show a correlation with task characteristics.

#### 3.2.3. Chen et al., 2019 [9]

EEG response to vibro-tactile stimulation are studied in younger and older adults addressing sensory capacity in order to clarify how age-related changes may affect the EEG signal and thus the use of BCI in older subjects. A total of 22 healthy individuals participated in the study, including 11 younger and 11 older adults. Authors analyzed event-related desynchronization/synchronization (ERD/S), i.e., the percentage of EEG power decrease/increase with respect to baseline, in order to explore cortical responses to left or right vibro-tactile stimulation. EEG signals were processed by CSP and LDA as classification algorithm. Results show decreased cortical lateralization of the somatosensory cortex and an overall reduction in EEG power in older subjects. This resulted in lower accuracy of BCI performance in classification based on spatial activation information.

#### 3.2.4. Zich et al., 2017 [10]

Age-related changes in the neural correlates of both motor execution (ME) and MI are studied, supported by EEG-based NF. A total of 37 healthy and cognitive preserved individuals participated in the study, including 19 younger and 18 older subjects. A multimodal neuroimaging system, focused on EEG event-related desynchronization (ERD%) and concentrations of oxygenated (HbO) and deoxygenated hemoglobin (HbR), was used in order to record simultaneously with functional near-infrared spectroscopy (fNIRS). Results of brain activity patterns show lower lateralization of ERD% and HbR concentration during MI, but not ME, in older subjects compared with younger participants. ERD% and hemodynamic measurements are significantly correlated, although there are no significant amplitude correlations.

#### 3.2.5. Hewerg et al., 2016 [11]

This work studies whether training improves tactile event-related potential (ERP)-BCI performance in a virtual wheelchair navigation task. A total of 10 healthy individuals participated in the study, with no control group. Older subjects participated in five sessions with calipers placed on the legs, abdomen, and back. The authors found that mean accuracy and information transfer rate (ITR) increased from the first session to the last session. The mean P300 amplitude went in the same direction, indicating improved performance thanks to training with a tactile P300-BCI. The protocol used enabled learning and significantly improved BCI performance (single trial accuracy and ITRs) and EEG features (ERP amplitudes, area between curves), demonstrating the positive effect of sensory training. Of particular note, authors found no plateau for ERP amplitudes, area between curves, single-trial accuracy, or ITRs, suggesting that participants could benefit from training. On the other hand, the effects of age-related changes in tactile perception on BCI performance appeared to be negligible.

#### 3.2.6. Gomez-Pilar et al., 2016 [12]

An NF training (NFT) with a motor imagery-based BCI (MI-BCI) was developed and used to analyze possible cognitive function improvements in 63 healthy older adults. To evaluate the effectiveness of NFT, the NFT group (31 subjects) that used this method and the control group (32 subjects) that did not perform NFT were compared. To investigate the effects of NFT, changes were analyzed both in the EEG spectrum using relative power measures and in various cognitive functions using the Luria Adult Neuropsychological Diagnosis (Luria- AND). The neuropsychological battery, which consists of nine subtests assessing different cognitive functions (i.e., visuospatial orientation, language, memory, reasoning, and attention), is administered before and after the NFT tasks. The between-group results show that significant differences are found between the control group and the NFT group in the Luria-AND values during the pretest. The within-group results show (1) in the control group, no significant changes in any of the cognitive functions and (2) in the NFT group, significant differences in all cognitive functions except attention.

#### 3.2.7. Karch et al., 2015 [13]

A supervised learning approach is developed to derive person-specific models for identifying and quantifying inter-individual differences in oscillatory EEG responses related to mechanisms for selecting and maintaining working memory (WM). A total of 52 healthy individuals participated in the study, including 20 children, 12 younger adults, and 20 older subjects. Differences between groups in rhythmic neural activity in the alpha frequency range were recorded, which were related to WM load and mechanisms for sustaining attention. Results show that WM load and spatial attentional focus could be distinguished in all age comparison groups based on EEG responses in the alpha range.

#### 3.2.8. Lee et al., 2015 [14]

Acceptability, safety, and preliminary efficacy are investigated for an EEG-based BCI cognitive training (CT) program in order to improve memory and attention in a Chinese-speaking group of older adults. To test the effectiveness of the CT, 39 cognitive preserved participants were randomly assigned to the intervention group (21 individuals) and the control group (18 individuals). Participants attended BCI-based CT sessions three times a week for 8 weeks; the control group received the same intervention after an 8-week waiting period. An adapted version of the Repeatable Battery for the Assessment of Neuropsychological Status (RBANS) was used to compare performance before and after training. This neuropsychological battery assesses several cognitive functions: memory, visual-spatial/constructive skills, language, and attention. All subjects were also asked to complete a usability and acceptability questionnaire, which included information on adverse events after each session. Results show statistically significant improvements in attention and delayed memory before and after CT. Conversely, not statistically significant changes are observed in immediate memory and visuospatial/constructive areas.

#### 3.2.9. Lee et al., 2013 [15]

The feasibility of the BCI system with a task that incorporates CT in improving memory and attention, previously used by the same authors in children with attention deficit hyperactivity disorder [18]. To test the effectiveness of the training in English-speaking group, 31 cognitive preserved older adults were randomly assigned to the intervention group (15 individuals) and the control group (16 individuals). Participants attended BCI-based CT sessions three times a week for 8 weeks; the control group received the same intervention after an 8-week waiting period. An adapted version of the Repeatable Battery for the Assessment of Neuropsychological Status (RBANS) was used to compare performance before and after training. This neuropsychological battery assesses several cognitive functions: memory, visual-spatial/constructive skills, language, and attention. All subjects were also asked to complete a usability and acceptability questionnaire, which included information on adverse events after each session. Results showed statistically significant improvements in immediate memory, visuospatial/constructive, attention, and delayed memory before and after CT.

## 4. Discussion

Aging induces a natural cognitive decline. Recent advances of technologies can help counteracting this process, keeping older people mentally active and performing. Specifically, EEG reflects cortical activity and can be used to monitor a subject during a mental task. Recent advances of technology is allowing flexible and real time applications (e.g., within the Internet of Things framework [19]) and artificial intelligence approaches (including machine learning, classification, deep learning, etc.) can extract useful information from EEG and provide a feedback to the user, within BCI and NF applications.

In this review, we selected specific works from the recent literature discussing innovative methods to support older people. The selected studies on innovative BCI technologies analyzed different domains of intrinsic capacity in terms of cognition [7,10,12,13,14,15], vitality [7], sensory [8,9,11], and locomotor capacities [8] to identify possible aging changes and the potential effectiveness of CT to promote and maintain healthy aging. They document that age-related differences are important to design properly a BCI [7,8,9,14,15] or a NF for stroke patients [10,12]. Moreover, training is important for BCI performance [11]. Differences are found between younger and older subjects, e.g., a reduced lateralization was documented in the latter [9,10]. However, all studies showed improvements in older subjects performing the training. Different protocols can be adopted: MI [7,10,12], motor control [8], tactile stimulation [9,11], or more complex mental activities [13,14,15].

A detailed discussion of the selected papers is provided below, by describing the domains of intrinsic capacity, giving priority to the works that took into consideration, primarily, cognition, and subordinately the other intrinsic capacities (that is, sensory and locomotor capacities).

- The study by Li et al. [7], which addressed intrinsic capacity (both cognition and vitality) during an MI task, showed that older people were less affected by the degree of cognitive fatigue, although the classification accuracy of the MI data was lower in older subjects compared to younger participants. Interestingly, the deep learning method, which extracts data from the frontal and parietal channels, may be appropriate for older individuals. Specifically, the authors found that classification accuracy on MI tasks was set by CNN at an acceptable level of about 70%. This suggests that the future prospects of BCI-MI in the older population need not to be based on SMR alone and that the appropriate algorithms can be applied without obvious lateralization of ERD. In fact, the CNN model based on fused spatial information greatly improves classification accuracy and leads to longer training time, which can be successfully used in healthy aging individuals. Therefore, it should be investigated whether these training sessions can support rehabilitation in aging people with neurological diseases, such as stroke patients.
- In the EEG-fNIRS study by Zich et al. [10], which focused on intrinsic capacity in relation to cognition, age-related changes in brain activity were analyzed in the neural correlates of both MI and ME. During MI, older adults showed lower hemispheric asymmetry of ERD% and HbR concentration than younger adults, reflecting greater ipsilateral activity. In addition, compared with no feedback, EEG-based NF-enhanced classification accuracy, thresholds, ERD% and HbR concentration for both contralateral activity and lateralization degree in both age groups. Finally, significant modulation correlations were found between ERD% and hemodynamic measurements, although there were no significant amplitude correlations. Overall, the differences between the observed effects for ERD%, HbR concentration and HbO concentration suggest that the relationship between electrophysiological motor activity and hemodynamics is far from clear. However, the results also support the idea that age-related changes in MI should be taken into account when designing MI NF protocols for patients. In particular, the influence of age should be carefully considered in the design of neuro-rehabilitation protocols for stroke patients. These results suggest a complex relationship between age and exercise-related activity in both EEG and hemodynamic measurements.
- In another study using the MI-BCI technology, Gomez-Pilar et al. [12], dealing with intrinsic capacity of cognition, showed promising results about the usefulness of NFT to improve brain plasticity and consequently neuropsychological functions (such as spatial awareness, language, and memory), which are the main concerns in older adults. This study may be helpful in the development of new NFT based on MI strategies. In particular, these data suggest the utility of BCI-based NFT in rehabilitating some cognitive functions in terms of improving brain plasticity, which seems to affect the older population.
- Karsch et al. [13] investigated inter-individual differences in brain-behavior mapping by examining the degree of model individualization required to demonstrate the feasibility of deriving person-specific models with different spatiotemporal information in three age groups (i.e., children, younger adults, and older adults). The authors focused on intrinsic capacity of cognition, i.e., mechanisms of selection and maintenance of working memory. The results show the potential of a multivariate approach to provide better discrimination than the classical non-person-specific models. Indeed, it allows easier interpretation at both individual and group levels to classify patterns based on rhythmic neural activity in the alpha frequency range across the lifespan. Specifically, information maintained at WM and the focus of spatial attention contributed to identify and quantify differences across age groups based on the different spatiotemporal properties of EEG recordings.
- Lee et al. [15] tested the potential of adapting an innovative computer-based BCI program for CT to improve attention and memory in a group of healthy English-speaking older adults. The authors, focusing on intrinsic capacity of cognition, demonstrated the effectiveness of the CT program, particularly in improving immediate and delay memory, attention, visuospatial, and global cognitive abilities. In a second randomized controlled trial [14], the same authors investigated the generalizability of their system and training task to a different language (i.e., Chinese-speaking) population of older adults. In their studies [14,15], the BCI-based intervention showed promising results in improving memory and attention. Future research should include participants with mild and severe cognitive impairment. If proven effective in a larger sample, this intervention could potentially serve to reduce, or even prevent, cognitive decline in patients with mild or major neurocognitive disorders.
- Chen et al. [9] addressed intrinsic-sensory capacity by examining whether SMR elicited by vibro-tactile stimulation shows differences in younger and healthy older adults. Their results showed that age-related electrophysiological changes significantly affect SMR properties. Specifically, older subjects showed less lateralization in somatosensory cortex in response to vibro-tactile stimulation compared to younger adults. These age-related EEG changes reflected greater susceptibility to noise and interference and resulted in lower BCI performance accuracy during classification. Future studies should focus on the effects of aging on EEG signals. In addition, NFT methods to improve cortical lateralization and algorithms not based solely on EEG lateralization should be investigated.
- Herweg et al. [11] investigated the effects of age and training in healthy older adults focusing on intrinsic sensory capacity and using a tactile stimulation protocol in a navigation task. Results showed that tactile BCI performance could be valuable, although age-related changes in somatosensory abilities were negligible. This protocol enabled learning and significantly improved BCI performance and EEG characteristics, demonstrating the positive effect of training. Future studies should focus on tactile BCI development, considering specific stimulation design, individual characteristics, and training. The results suggest that tactile BCIs can not only be a valid alternative to visual and auditory tasks, but can also be used despite age-related changes in somatosensory abilities.
- Goelz et al. [8] investigated intrinsic-sensory and locomotor capacities. The authors analyzed the classification of fine motor movements in terms of age-related differences in functional brain activity. Specifically, the authors compared the performance of younger and older healthy adults on visuomotor tracking tasks using EEG recordings. Results revealed electrophysiological brain activity patterns associated with an altered sensorimotor network in older adults, suggesting reorganization of task-related brain networks in response to task features. Future research on BCI applications should consider age-related differences in the development of BCI and neurofeedback systems when targeting the older population (e.g., in the selection of appropriate features and algorithms).

In summary, the selected studies showed age-related differences as important features for the design of BCI technologies [7,8,9,10,13] or the effectiveness of NFT in improving brain plasticity and some neuropsychological functions [10,12]. In addition, CT [14,15] and tactile [11] training are important for BCI performance.

We note as a limitation that different protocols were used in the included studies, such as MI [7,10,12], motor control [8], tactile stimulation [9,11], or more complex mental tasks [13,14,15] and focused on different domains (e.g., cognition, vitality, sensory, and motor skills). These aspects did not allow us to adequately compare the collected data to better understand age-related changes that could help improve the knowledge of BCI technologies to develop new healthcare solutions for the older population. Thus, there is a clear need to explore this important topic further in the future and to standardize the investigation techniques.

The perspective presented in our systematic review may lead to new challenges and promising results in the detection of possible age changes in healthy individuals and in the rehabilitation of patients with physical and cognitive impairments whose intrinsic capacity may be impaired due to disease. Following the theoretical approach based on the World Health Organization guidelines for healthy aging [1], future studies may be helpful to demonstrate the effectiveness of rehabilitation treatments developed with new specific technologies and devices based on BCI, such as those that support and improve cognitive and physical abilities.

From the comprehensive person-centered perspective proposed in [1], “functional capacity” combines the interactions between a person’s physical and mental abilities (intrinsic capacity) and the living environment. Considering this paradigm and the characteristics of intrinsic capacity (locomotor and sensory capacity, vitality, and cognition), interventions with BCI provide useful support for older people in their daily activities.

In particular, interventions with BCI provide useful support for maintaining and achieving healthy aging, as shown by the studies analyzed in our review.

Regarding patients, neuro-rehabilitative BCI applications offer interesting perspectives, for example, in terms of cognitive and mental abilities of Alzheimer’s disease patients, mobility, and fine motor skills of patients with Parkinson’s disease, or physical abilities of patients with amyotrophic lateral sclerosis. In other neurological patients, BCI technologies may be useful to improve communication/control, e.g., motor paralysis due to stroke, spinal cord injury, or cerebral palsy [3].

## 5. Conclusions

Artificial intelligence approaches applied to EEG are useful to provide feedback to the user in BCI and NF applications. In fact, EEG, reflecting cortical activity, can be used to monitor a person during a mental task, which can be decoded by artificial intelligence to provide the required feedback.

Recent advances in BCI technologies can help counteract the aging process and keep older people healthy and active. Our systematic review, based on PRISMA guidelines, employed strict selection criteria (i.e., innovative works on HMI in older adults using EEG signals). The selected studies were helpful in identifying possible age-related changes and the potential effectiveness of CT.

Future studies using BCI technology would help understand how a person develops and maintains intrinsic abilities, to promote healthy aging and reduce the occurrence of unfavorable aging trajectories. Finally, the use of HMI could help physically and cognitively impaired individuals to support basic activities of daily living and reduce dependence on care. The few but promising works identified testify to the need for further development of EEG-based HMIs to counteract age-related decline.

## Figures and Tables

**Table 1:** Participants’ characteristics in the included studies. Notation: **NP** = neuropsychological; **M** = mean; **SD** = standard deviation.

Reference	Number of Participants	Females/Males (%)	Age Range	Age in Years M (±SD)	NP Assessment	Control Group
Li et al., 2022 [7]	20	70/30		66	no	Younger adults
Goelz et al., 2021 [8]	26	61.5/38.5	55–65		no	Younger adults
Chen et al., 2019 [9]	22	72.7/27.3	over 55	72 (±8.1)	no	Younger adults
Zich et al., 2017 [10]	37	55.6/44.4		62.6 (±5.7)	yes	Younger adults
Herweg et al., 2016 [11]	10	60/40	50–73	60 (±6.7)	no	no
Gomez-Pilar et al., 2016 [12]	63	65.1/34.9	60–81		yes	Older adults without training
Karch et al., 2015 [13]	52		70–75	73.3	yes	Children and younger adults
Lee et al., 2015 [14]	39	69.2/30.8		65.2(±2.8)	yes	Older adults without training
Lee et al., 2013 [15]	31	60/40		65.1 (±2.9)	yes	Older adults without training

**Table 2:** Selected studies taking into account intrinsic capacity evaluation and processing. FBCSP: filter bank CSP. SWLDA: step-wise LDA.

Reference	Intrinsic Capacity	BCI Assessment	Spatial Filtering	Feature Processing	Classification (Translation)
Li et al., 2022 [7]	Cognition and Vitality	Motor imagery	ICA		CNN
Goelz et al., 2021 [8]	Sensory and Locomotor	Visuo-motor task	ICA	FBCSP	LDA
Chen et al., 2019 [9]	Sensory	Vibro-tactile	CSP	CSP	LDA
Zich et al., 2017 [10]	Cognition	Motor imagery	CSP		LDA
Herweg et al., 2016 [11]	Sensory	Tactile sensibility		ERP	SWLDA
Gomez-Pilar et al., 2016 [12]	Cognition	Motor imagery	ICA, Laplacian	Power of EEG rhythms	Power of EEG rhythms
Karch et al., 2015 [13]	Cognition	Working memory performance	CSP		LDA
Lee et al., 2015 [14]	Cognition	Cognitive training program	CSP	Power of EEG rhythms	Score based on CSP and PSD
Lee et al., 2013 [15]	Cognition	Memory and attention training program	CSP	Power of EEG rhythms	Score based on CSP and PSD

## Data Availability

Not applicable.

## Supplementary Materials

The following supporting information can be downloaded at: https://www.mdpi.com/article/10.3390/bioengineering10040395/s1, Table S1. Excluded studies and reasons for the exclusion. Table S2. Results and perspectives of the papers fitting our selection criteria.

## Abbreviations

The following abbreviations are used in this manuscript:

ALS	amyotrophic lateral sclerosis
ANN	artificial neural network
ApEn	approximate entropy
AR	autoregressive
ARMA	autoregressive moving average
ARV	average rectified value
BCI	brain–computer interface
BSS	blind source separation
CNN	convolutional neural networks
CSP	common spatial pattern
CT	cognitive training
EEG	electroencephalogram
FIR	finite impulse response
HMI	human–machine interaction
ICA	independent component analysis
IIR	infinite impulse response
ITR	information transfer rate
LDA	linear discriminant analysis
LLE	local linear embedding
MA	moving average
MI	motor-imagery
NF	neurofeedback
NFT	neurofeedback training
PAC	phase amplitude coupling
PCA	principal component analysis
PLV	phase locking value
PRISMA	Preferred Reporting Items for Systematic Review and Meta-Analyses
PSD	power spectral density
RBANS	Repeatable Battery for the Assessment of Neuropsychological Status
RMS	root mean squared
SNR	signal to noise ratio
SVM	support vector machine
TFR	time–frequency representations
WM	working memory

## Appendix A. Background on Data Processing and Classification

A short general and basic introduction to the processing methods which are most popular in applications is provided. Elements of signal conditioning, detection, pre-processing, processing, and classification are discussed, focusing on the EEG. Many didactic textbooks and surveys are available on these general topics: signal processing [20,21], machine learning [22], classification [23], and applications [24].

### Appendix A.1. Signal Acquisition

Physiological data are detected by sensors. We are mostly interested to surface EEG, so that the physiological data to be recorded refer to the cortical activity and the sensors are electrodes placed on the scalp. The signal is detected by a standard cascade of systems that include an amplifier with high input impedance (which should be much higher than that of the skin-electrode contact to avoid voltage drop), a filter and an AD converter. The filter allows only specific frequency components to pass, which is useful to reduce the amount of recorded noise and to satisfy the requirements of sampling theory. Signal decomposition into frequency components and sampling theory are introduced in the following sections.

### Appendix A.2. Fourier Analysis

Every periodic signal can be written as a Fourier series, i.e., the sum of a constant and sinusoids with frequencies, in multiples of the fundamental frequency (i.e., the reciprocal of the period) of the original signal. If the signal x(t) is not a periodic function of time *t*, we can make a limit as the period that reaches infinity [21], obtaining the Fourier transform, in which the sum of the components of the Fourier series is substituted by an integral and the frequency becomes a continuous variable

(A1) $$X\left(f\right)=\int_{-\infty }^{+\infty }x\left(t\right)e^{-j2\pi ft}dt$$

where *f* is the frequency and the sinusoids mentioned above arise from the complex exponential $e^{-j2\pi ft}=\cos\left(2\pi ft\right)-j\sin\left(2\pi ft\right)$ (Euler formula). The Fourier transform X(f) indicates for each frequency the amplitude and the phase of the corresponding sinusoidal component included in the signal x(t). By summing (through an integral) all those components, the following inversion formula is obtained

(A2) $$x\left(t\right)=\int_{-\infty }^{+\infty }X\left(f\right)e^{j2\pi ft}df$$

These relations hold for all signals of interest (e.g., those with finite energy) and indicate a one-to-one correspondence so that our specific signal can be studied either in the time or in the frequency domain.

In practice, the measurement is limited in time so that we cannot have access to the original signal x(t) (which is unknown out of the time range of observation), but to a perturbed version of it, obtained by a multiplication with an observation window, selecting the data that have been acquired and putting to zero the rest. This perturbation introduces some problems in the investigation of the Fourier transform of the original signal and some limitations (e.g., we cannot observe very slow frequency components with periods longer than the observation window).

Fourier transform is a complex function of frequency (in the sense that it includes both a real and an imaginary part). Usually, only its power spectral density (PSD, i.e., the modulus squared of the Fourier transform) is considered. When signals have properties which change in time, i.e., they are not stationary, the PSD is not sufficient to characterize them accurately, as it indicates only which frequency components are included, but not when they are present. Time–frequency representations (TFR) have been introduced to spit the signals into frequency components, indicating their power density as a function of time and frequency [25]. An interesting alternative is using time-scale representations, i.e., wavelet transform [26], in which the signal is decomposed into components which are scaled versions of a specific waveform (mother wavelet, which has a finite time support, as opposite to sinusoids).

When processing the signal by a linear system, superposition principle holds. It means that different components can be studied separately (e.g., by amplifying some or attenuating others) and the output can be obtaining by summing up. Classical processing of signals by removing specific components is called filtering: lowpass, bandpass, highpass, and stopband filters remove frequency components out of the range of interest (indicated by the name), which is left ideally unaltered. A value separating the pass- from the stop-band is called cutoff frequency (more cutoff frequencies are defined in cases in which more boundaries between passband and attenuated regions are present, e.g., for the bandpass filter).

### Appendix A.3. Sampling Theory

Physiological data are sampled with a constant sample rate $f_{s}$, converting them in digital form x[n]=x(nT), where $T=1/f_{s}$ is the sampling interval. The Fourier transform of the sampled data is a periodic repetition of that of the original signal, with period $f_{s}$. The repetitions, called alias (from Latin), give high frequency components (aliasing phenomenon) that should be removed to obtain the original signal from its sampled version, using a lowpass filter with cutoff frequency lower than or equal to $f_{s}/2$. The latter is called Nyquist frequency and is the largest one that can be represented in a sampled signal: it requires that two samples are acquired for each cycle of the component with largest frequency. With a smaller sampling rate, the component would be confused with another of lower frequency, contributing to aliasing. For this reason, before sampling, it is better to remove components with frequency larger than Nyquist frequency by a so-called anti-aliasing lowpass filter.

### Appendix A.4. Filtering

EEG usually has useful information embedded within a great amount of noise. A quantitative measure of this challenging condition is given by the signal to noise ratio (SNR), which is the ratio between the energy of the signal and that of noise (usually measured in dB). In order to increase the SNR, a pre-processing is applied through filtering.

Data are acquired by a sampling in time (discussed in the previous section) and space (as a discrete distribution of electrodes are placed over the scalp). By combining those samples, specific filters can be obtained, in order to enhance the components of interest and reduce the effect of artifacts or noise.

#### Appendix A.4.1. Temporal Filters and EEG Rhythms

Filtering in time is applied separately to each recorded signal, by defining the output time series y[n] as the linear combination of the input signal x[n] at different time delays and previous values of the output

(A3) $$y\left[n\right]=b_{0}x\left[n\right]+b_{1}x\left[n-1\right]+\;\ldots \;+b_{k}x\left[n-k\right]-a_{1}y\left[n-1\right]-\;\ldots \;-a_{m}y\left[n-m\right]$$

In this way, only present and past samples are used, obtaining a causal filter (which means that no future sample is used to define the present output). If only input data are used to define the output (i.e., all weights $\left\{a_{i}\right\}$ are zero), the filter is said moving average (MA), as the same weighted average of samples is applied to subsequent portions of input data. The weights constitute the samples of the so-called impulse response h[n] of the filter. They are the output of the filter if the input is an impulse (i.e., a digital signal with a single value equal to 1 and zero otherwise). The operation of multiplying the signal by the impulse response translated in time and summing is called convolution

(A4) $$y\left[n\right]=\sum_{k=-\infty }^{+\infty }h[n-k]\;x[k]$$

The use of past values of the output in the definition of the filter is called autoregression (AR). By iterative substitution of the definition of delayed output values y[n−p] in terms of inputs x[n−q] (p≤m, q≤k) we see that the impulse response is infinite for AR and ARMA filters (i.e., those including only AR or both AR and MA filtering), in the sense that it has infinite samples. This allows improve filtering performances over MA filters, but with the risk of obtaining unstable results, as the convolution (A4) includes infinite terms and could diverge. The terms finite impulse response (FIR) and infinite impulse response (IIR) are used to denote these two classes of filters (i.e., MA is a FIR filter; AR and ARMA are IIR filters).

By choosing properly the weights of the filter, it is possible to obtain approximations of classical filters selecting specific frequency components to pass and others to be attenuated. This way, we can perform important processing such as slow trend attenuation (by a highpass filter), high frequency noise reduction (lowpass filter), powerline artifact removal (selective stopband or notch filter), or EEG rhythm extraction (bandpass filter).

There are also temporal filters that are adapted to the signal to be processed. The match filter has an impulse response that is equal to a template of interest to be found within the signal (except for a flip of the time variable). The convolution (A4) computes the correlation between the template and the signal (as a function of the time shift of the template), which is high when a waveform similar to the template is found.

Another popular method, called averaging, is applied to extract evoked responses from the noisy EEG: epochs of EEG centered on the event triggering the response are extracted from the EEG trace and averaged; the evoked response is reinforced whereas the asynchronous contributions (due either to noise or to other cortical activities) are reduced.

#### Appendix A.4.2. Spatial Filters and Selectivity

The signals from different electrodes can be linearly combined, making a spatial filter. Different objectives can be achieved by spatial filtering. For example, a common mode potential is usually present due to a capacitive coupling of the body with the powerline. Moreover, due to the volume conduction, the potential reflecting the activity of a cortical source spreads over the scalp, providing contributions to the recording of many electrodes and reducing the possibility of localizing its position. Spatial filters can remove this common mode potential by imposing that the sum of the weights is zero. For example, the average surface potential recorded from all the electrodes can be used as reference (average reference method), eliminating the common mode. Different combinations have also been proposed, which are discrete versions of differential operators: the bipolar (i.e., the difference of signals from close electrodes) approximates the first derivative (which can be taken either in longitudinal, i.e., cranio-caudal, or transverse direction), the double differential (difference of two consecutive bipolar filters with the central electrode in common) is the discrete version of the second derivative, the Laplacian filter (sum of longitudinal and transverse double differential) resembles the Laplacian operator. They have better selectivity with respect to an unipolar detection (i.e., from a single electrode): this means that they are focused on the cortical activity which is under the center of the filter (said in an alternative way, they have a smaller detection volume).

As an alternative to the above-mentioned filters, which have fixed weights, there are others which are defined on the basis of the signal, in order to obtain specific objectives.

1. Principal component analysis (PCA [27]) chooses the weights in order to obtain output signals that are orthogonal (i.e., uncorrelated) to each other.
2. Independent component analysis (ICA [27]) selects linear combinations that makes the outputs statistically independent from each other.
3. Common spatial pattern (CSP [28]) is obtained by optimizing the filter in order to separate the signal into the sum of components which show maximum difference of the variances between two windows. An application is in enhancing the energy of a specific target portion of data (e.g., the cortical potential of interest for a BCI) and reducing that of the rest.

PCA and ICA found many applications to separate blindly different sources (blind source separation—BSS) and to compress the data (by selecting only the most important components contributing for a certain amount of energy or information included in the data). Among the components that are identified, some could be due to artifacts (e.g., blinks or muscle contractions) and can be removed.

### Appendix A.5. Feature Processing and Classification

The above-mentioned method can be the basis for extracting useful information from EEG data. Then, this information could be translated to some target output of interest, indicating the condition of the user or a choice among different options.

Here, we briefly introduce how EEG features can be extracted and used to obtain information on the user. Those features can be obtained by processing either directly the EEG signals or source data. The latter are obtained by estimating the neural sources generating the recorded EEGs, by solving a complicated inverse problem [29]. However, in the following, we refer to processing directly the recorded EEG channels.

#### Appendix A.5.1. Feature Extraction

Different features can be estimated from each EEG channel, represented either in time or in frequency domain.

- Time features can be the average rectified value (ARV) or the root mean squared (RMS) of epochs of data, the height of peaks of evoked potentials, the number of zero-crossings. More advanced features reflect the possible complexity of the data: for example, approximate entropy (ApEn [30]), and fractal dimension [31].
- Interesting features extracted from a frequency representation of the signal are the mean frequency, the median frequency or the power of different rhythms.

The topographical distribution of an index can provide useful information, e.g., on a possible asymmetry across the hemispheres, or on specific responses (for example, the amplitude of alpha rhythm has different meanings when found in occipital or frontal regions).

Moreover, bivariate indexes have been introduced to investigate the coupling of different regions, i.e., the functional connectivity reflected by EEG [32]. Examples of indexes are listed below.

1. Cross-correlation quantifies a linear coupling of the EEGs from two channels. The delay corresponding to the maximal cross-correlation can provide also some insight on the causal relationship between the two signals.
2. Coherence provides information on correlation of oscillations as a function of their frequency.
3. Nonlinear indexes indicate couplings at a statistical order larger than linear. For example, mutual information [33] and transfer entropy [34] are based on Shannon information theory and are zero for statistically independent inputs. Nonlinear interdependence is based on the study of possible coupling during recurrences, i.e., when the data show similar behavior [35].
4. Phase locking value (PLV) detects periods in which the phase delay of oscillations from different channels remains constant (so that the oscillations are locked) [36].
5. Granger causality indicates a causal-effect relation between the EEGs from two channels, given in terms of the improved performance in predicting future samples of a signal when information from the other is provided [37].

Bivariate indexes can be defined for each pair of EEG channels and their values can be collected in an adjacency matrix. The information from this amount of data can be summarized by topological indexes from graph theory [38]. A graph is a set of nodes (i.e., EEG channels in our case) connected by edges (i.e., the bivariate indexes coupling pairs of nodes). Many indexes have been proposed to characterize a graph, e.g., strength (sum of the connections to a node), clustering (degree to which nodes in a graph cluster together), efficiency (ability to exchange information), and characteristic path length (measure of how information is transferred across the network).

Cross-frequency coupling explores possible relations between different rhythms [39]. It can be studied either within or between channels, obtaining univariate and bivariate information, respectively. An example is the phase amplitude coupling (PAC), which measures the relation between the amplitude envelop of a high-frequency rhythm (e.g., beta or gamma) and the phase of a low-frequency rhythm (e.g., alpha).

#### Appendix A.5.2. Feature Generation

Features can be either used directly or post-processed, generating other features. For example, when extracting many features from the EEG, some of them can be redundant and share common information. Moreover, features can be noisy. Thus, methods of dimensionality reduction, such as the above-mentioned methods, PCA and ICA, are useful also to process features: they can cancel out common information and select a few components representing most of the useful content and attenuate noise. PCA and ICA are linear methods, i.e., their components are linear combination of the inputs. Other alternative methods have also been introduced to reduce the dimension of the dataset (thus summarizing the information they contain) by a nonlinear processing, e.g., the local linear embedding (LLE [40]).

All mentioned approaches are only based on the input data. Other methods process the input data to help performing the classification. For example, the linear discriminant analysis (LDA [41]) provides components (defined as linear combinations of the features) that allows optimal discrimination of different output classes (in the sense that the between- and within-class separation of data points are maximized and minimized, respectively).

#### Appendix A.5.3. Feature Selection

Not all features could be important to perform the classification, as there could be redundancy among them, some could be irrelevant or they could even cause misclassification. The goals of features selection are:

- Avoiding overfitting;
- Reducing the computational cost;
- Gaining a deeper insight into the classification/prediction model.

Criteria for feature selection can be either based on intrinsic properties of the features (filtering methods, as selecting components with high energetic or information content, provided by PCA and ICA, respectively) or on their contribution in improving the performances of the classifier (wrapped approaches, e.g., measuring features providing maximal information on the output class and with minimal redundancy among them, or sequentially selecting/removing the most/least influential feature in the classification of a training dataset).

#### Appendix A.5.4. Classification

The features obtained after the previous steps provide some indirect information on the user’s intent, which should be translated to a target output, i.e., a command to a device which could provide, for example, a feedback to the user, or the selection of an answer to a question, or the displacement of a cursor to a screen [24]. The classification (or translation) of input data into a useful command can be interpreted as the definition of a mathematical model mapping input observations (i.e., vectors of features) into a specific target output [23].

The model has parameters to be optimally chosen to obtain the output of interest. Some training data (i.e., EEG features associated to known labeled outputs) are used in supervised learning, in which the model parameters are selected to fit the output of interest in terms of an optimization criterion (e.g., the minimization of the mean squared difference between corrected output and model prediction).

Once fitted the training data, the model can be applied on test samples. The reliability of the predictor on new test data is called generalization. There is a trade-off between an optimal fitting of the training data (requiring a complex model) and generalization (as simpler models are more robust, i.e., they have similar performances in the training and test data). Thus, the simpler model allowing to decode the determinism contained into the training data should be selected, in order to avoid to fit also the noise, which would provide unstable and unpredictable behavior when testing new data. Different criteria have been proposed to balance model fitting performance and complexity (e.g., Akaike information criterion [42]). As an alternative, a portion of the training set could be used for validation, in order to test the performance of the model in an external set (i.e., not used for training); for example, in cross-validation, different portions of data are used to train and validate the model in different iterations.

Different general classification problems are the identification of:

- A classification function to discriminate between discrete choices,
- A regression model to translate input observations into a continuous variable.

Many classification or regression models have been proposed in the literature. Some of them are introduced below.

- Linear regression [43]. The output *Y* is approximated as a linear combination of the inputs ${\left\{X_{i}\right\}}_{i=1,\cdots ,N}$ (*N* being the number of input features)
  (A5) $$Y=w_{0}+w_{1}X_{1}+\cdots +w_{N}X_{N}$$
  where the weights ${\left\{w_{k}\right\}}_{k=0,\cdots ,N}$ are chosen optimally with respect to some criterion. For example, imposing the minimum sum of squared error (considering all observations), the weights can be obtained analytically by pseudoinversion of the matrix *A* including the features
  (A6) $$A=\left[\begin{array}{cccc} 1 & x_{11} & \cdots & x_{N1}\\ 1 & x_{12} & \cdots & x_{N2}\\ \vdots & \vdots & \vdots & \vdots \\ 1 & x_{1M} & \cdots & x_{NM} \end{array}\right]$$
  where *M* is the number of observations and ${\left\{x_{ij}\right\}}_{i=1,\cdots ,N;j=1,\cdots ,M}$ are the elements of the *i*th feature $X_{i}$. The problem can be written in matrix form as
  (A7) Y=AW
  where *W* is the vector including all weights. The solution with minimum squared error is
  (A8) $$W={\left(A^{T}A\right)}^{-1}A^{T}Y$$
  where ${\left(A^{T}A\right)}^{-1}A^{T}$ is the pseudoinverse of *A* (notice that $A^{T}Y$ is the cross-correlation of the input features and desired output, $\left(A^{T}A\right)$ represents the covariance between features).
  A linear regression problem is also obtained if a polynomial expression or another nonlinear function of the features is included as additional input to better fit the data: more columns are included in the matrix *A*, but we still obtain a linear problem with respect to the weights.
- Bayesian classifiers [43]. A probabilistic model is built based on the training data to evaluate the probability of the input feature conditional to specific outputs P(XY). Then, a priori information on the output probabilities P(Y) allows to obtain the posterior probability of the output given the inputs, using Bayes theorem
  (A9) $$P\left(YX\right)=\frac{P(XY)P(Y)}{P(X)}$$
  The estimated output in the test set is given by the one with maximal posterior probability.
- Classification tree [44]. Decision trees are predictive models in which target variables are the leaves of a tree. They can take a finite set of output values. They are multistage systems, in which classes are rejected sequentially, until an accepted class is arrived upon. Specifically, the feature space is sequentially split into clusters, corresponding to classes. A set of questions is applied to individual features, which are compared to threshold values, on the basis of which the decision tree branches.
  Different approaches have been proposed to select the features and associated thresholds to split data until reaching a decision on the estimated class.
- Support vector machine (SVM) [45]. A hyper-surface is searched that best separates two classes (in the multi-class case, a combination of binary classifications is used). Support vectors are specific observations (i.e., points in the space of input features) at the border between two classes that define the lower and upper margins of a region separating the two classes. This hyper-surface separating the two classes is chosen in order to maximize the margin.
- Neural network [46]. An artificial neural network (ANN) is a biologically inspired computational model consisting of a complex set of interconnections between basic computational units, called neurons. Each neuron performs a nonlinear processing, by a so-called activation function applied on the sum of a bias and a linear combination of the input. The bias and the weights of the linear combinations defining the input of each neuron of the network are the parameters to be selected to obtain a specific objective, e.g., minimizing the mean-squared error in estimating the target output on a training dataset.
  Different topologies of the networks have been considered, e.g., multi-layer perceptron (with a forward structure of sequential layers of neurons, with each layer obtaining input from the previous one and providing output to the following one) with a different number of layers, recursive networks (with the output of one layer of neurons providing inputs to a previous layer).
  Different learning algorithms have been proposed: backpropagation or other methods related to the gradient of the objective functional; evolutionary algorithms (e.g., genetic approaches or particle swarm optimization) designed to prevent being stuck into a local minimum of the error function.
  Deep learning approaches have been recently proposed showing exceptional performances [47]. They are based on networks with many layers. Some of these layers can perform a convolution operation (convolutional neural networks—CNN) with a kernel adapted to the data.

#### Appendix A.5.5. Performance Evaluation

An important problem of a classifier is generalization, i.e., the ability of working properly on data which were not used for training. The generalization error has two main components.

1. Bias, related to the ability of the classifier to approximate the true model generating the data. It is due to inaccurate assumptions or simplifications made by the classifier.
2. Variance, quantifying how much classifiers estimated from different training sets differ from each other.

When the assumed model is too simple to represent all the relevant information contained in the data, there is a high bias and low variance, with high training and test errors, reflecting an underfitting of the true model generating the data. On the other hand, there is overfitting when the model is too complex and fits irrelevant characteristics (noise) in the data. In this case, the classifier shows low bias and high variance, with low training error and high test error.

The best classification model should have a complexity which is in between the conditions of underfitting and overfitting, so that to decode the determinism contained in the data without fitting noisy perturbations. It is chosen either using a specific criterion to be minimized, weighting both the error in fitting the data and the complexity (e.g., Akaike information criterion [42]), or by separating the training data into two subsets (called training and validation sets) in order to test directly the generalization of the classifier on data not used for training (i.e., the validation set). Two methods of this latter type are the cross-validation and Monte Carlo approaches.

Cross-validation is based on the following steps.

1. Split training data into *k* equal parts.
2. Train the model on k−1 parts and calculate validation error on the *k*th one.
3. Repeat *k* times, using each data subset for validation once.

Monte Carlo approach requires the following steps.

1. Split training data randomly.
2. Fit the model on training data and calculate the validation error.
3. Repeat for many iterations (say 100 or 500) and take the average of the validation errors.

By these methods, the best classifier can be selected and its performances can be estimated on a test set.

## References

[1] World Health Organization (2020). Decade of Healthy Ageing: Baseline Report. https://apps.who.int/iris/handle/10665/338677.

[2] Jiang Y., Abiri R., Zhao X. (2017). Tuning up the Old Brain with New Tricks: Attention Training via Neurofeedback. Front. Aging Neurosci..

[3] Belkacem A.N., Jamil N., Palmer J.A., Ouhbi S., Chen C. (2020). Brain Computer Interfaces for Improving the Quality of Life of Older Adults and Elderly Patients. Front. Neurosci..

[4] Belkacem A.N., Falk T.H., Yanagisawa T., Guger C. (2022). Editorial: Cognitive and Motor Control Based on Brain-Computer Interfaces for Improving the Health and Well-Being in Older Age. Front. Hum. Neurosci..

[5] Page M.J., McKenzie J.E., Bossuyt P.M., Boutron I., Hoffmann T.C., Mulrow C.D., Shamseer L., Tetzlaff J.M., Akl E.A., Brennan S.E. (2021). The PRISMA 2020 statement: An updated guideline for reporting systematic reviews. BMJ.

[6] World Health Organization (2022). Health Topics, Ageing. https://www.who.int/health-topics/ageing#tab=tab_1.

[7] Li X., Chen P., Yu X., Jiang N. (2022). Analysis of the Relationship Between Motor Imagery and Age-Related Fatigue for CNN Classification of the EEG Data. Front. Aging Neurosci..

[8] Goelz C., Mora K., Rudisch J., Gaidai R., Reuter E., Godde B., Reinsberger C., Voelcker-Rehage C., Vieluf S. (2021). Classification of visuomotor tasks based on electroencephalographic data depends on age-related differences in brain activity patterns. Neural Netw..

[9] Chen M.L., Fu D., Boger J., Jiang N. (2019). Age-Related Changes in Vibro-Tactile EEG Response and Its Implications in BCI Applications: A Comparison Between Older and Younger Populations. IEEE Trans. Neural Syst. Rehabil. Eng..

[10] Zich C., Debener S., Thoene A.K., Chen L.C., Kranczioch C. (2017). Simultaneous EEG-fNIRS reveals how age and feedback affect motor imagery signatures. Neurobiol. Aging.

[11] Herweg A., Gutzeit J., Kleih S., Kübler A. (2016). Wheelchair control by elderly participants in a virtual environment with a brain-computer interface (BCI) and tactile stimulation. Biol. Psychol..

[12] Gomez-Pilar J., Corralejo R., Nicolas-Alonso L.F., Álvarez D., Hornero R. (2016). Neurofeedback training with a motor imagery-based BCI: Neurocognitive improvements and EEG changes in the elderly. Med. Biol. Eng. Comput..

[13] Karch J.D., Sander M.C., von Oertzen T., Brandmaier A.M., Werkle-Bergner M. (2015). Using within-subject pattern classification to understand lifespan age differences in oscillatory mechanisms of working memory selection and maintenance. NeuroImage.

[14] Quek S.Y., Lee T.-S., Goh S.J.A., Phillips R., Guan C., Cheung Y.B., Feng L., Wang C.C., Chin Z.Y., Zhang H.H. (2015). A pilot randomized controlled trial using EEG-based brain-computer interface training for a Chinese-speaking group of healthy elderly. Clin. Interv. Aging..

[15] Lee T.-S., Goh S.J.A., Quek S.Y., Phillips R., Guan C., Cheung Y.B., Feng L., Teng S.S.W., Wang C.C., Chin Z.Y. (2013). A brain-computer interface based cognitive training system for healthy elderly: A randomized control pilot study for usability and preliminary efficacy. PLoS ONE.

[16] Massetti N., Russo M., Franciotti R., Nardini D., Mandolini G.M., Granzotto A., Bomba M., Pizzi S.D., Mosca A., Scherer R. (2022). A Machine Learning-Based Holistic Approach to Predict the Clinical Course of Patients within the Alzheimer’s Disease Spectrum. J. Alzheimer’S Dis. JAD.

[17] Luo J., Sun W., Wu Y., Liu H., Wang X., Yan T., Song R. (2018). Characterization of the coordination of agonist and antagonist muscles among stroke patients, healthy late middle-aged and young controls using a myoelectric-controlled interface. J. Neural Eng..

[18] Lim C.G., Lee T.S., Guan C.T., Fung D.S., Zhao Y., Teng S.S., Zhang H., Krishnan K.R. (2012). A brain-computer interface based attention training program for treating attention deficit hyperactivity disorder. PLoS ONE.

[19] Kumar D.P., Srirama S.N., Amgoth T., Annavarapu C.S.R. (2021). Survey on recent advances in IoT application layer protocols and machine learning scope for research directions. Digit. Commun. Netw..

[20] Marple S.L. (1987). Digital Spectral Analysis with Applications.

[21] Mesin L. (2017). Introduction to Biomedical Signal Processing.

[22] Kumar D.P., Amgoth T., Annavarapu C.S.R. (2019). Machine learning algorithms for wireless sensor networks: A survey. Inf. Fusion.

[23] Theodoridis S., Koutroumbas K. (2008). Pattern Recognition.

[24] Wolpaw J., Winter Wolpaw E. (2012). Brain–Computer Interfaces: Principles and Practice.

[25] Cohen L. (1989). Time-Frequency Distributions—A review. Proc. IEEE.

[26] Rioul O., Vetterli M. (1991). Wavelets and signal processing. IEEE Signal Process. Mag..

[27] Mesin L., Holobar A., Merletti R., Cerutti S., Marchesi C. (2011). Blind source separation: Application to biomedical signals. Advanced Methods of Biomedical Signal Processing.

[28] Koles Z.J., Lazaret M.S., Zhou S.Z. (1990). Spatial patterns underlying population differences in the background EEG. Brain Topogr..

[29] Jatoi M.A., Kamel N., Malik A.S., Faye I., Begum T. (2014). A Survey of Methods Used for Source Localization Using EEG Signals. Biomed. Signal Process. Control..

[30] Mesin L. (2018). Estimation of Complexity of Sampled Biomedical Continuous Time Signals Using Approximate Entropy. Front. Physiol..

[31] Mandelbrot B. (1967). How Long is the Coast of Britain? Statistical Self-Similarity and Fractional Dimension. Science.

[32] Park H., Friston K. (2013). Structural and functional brain networks: From connections to cognition. Science.

[33] Ibáñez-Molina A.J., Soriano M.F., Iglesias-Parro S. (2020). Mutual Information of Multiple Rhythms for EEG Signals. Front. Neurosci..

[34] Schreiber T. (2000). Measuring information transfer. Phys. Rev. Lett..

[35] Arnhold J., Grassberger P., Lehnertz K., Elger C.E. (1999). A robust method for detecting interdependences: Application to intracranially recorded eeg. Phys. D.

[36] Lachaux J., Rodriguez E., Martinerie J., Varela F. (1999). Measuring phase synchrony in brain signals. Hum. Brain Mapp..

[37] Granger C.W.J. (1969). Investigating Causal Relations by Econometric Models and Cross-Spectral Methods. Econometrica.

[38] Rubinov M., Sporns O. (2010). Complex network measures of brain connectivity: Uses and interpretations. Neuroimage.

[39] Hyafil A., Giraud A.-L., Fontolan L., Gutkin B. (2015). Neural cross-frequency coupling: Connecting architectures, mechanisms, and functions. Trends Neurosci..

[40] Roweis S.T., Saul L.K. (2000). Nonlinear Dimensionality Reduction by Locally Linear Embedding. Science.

[41] Xanthopoulos P., Pardalos P.M., Trafalis T.B. (2013). Linear Discriminant Analysis. Robust Data Mining.

[42] Akaike H. (1974). A new look at statistical model identification. IEEE Trans. Autom. Control..

[43] Rencher A.C., Schaalje G.B. (2008). Linear Models in Statistics.

[44] Quinlan R., Michalski R.S., Carbonell J.G., TMitchell M. (1983). Learning efficient classification procedures. Machine Learning: An Artificial Intelligence Approach.

[45] Cortes C., Vapnik V. (1995). Support-vector networks. Mach. Learn..

[46] Haykin S. (1999). Neural Networks: A Comprehensive Foundation.

[47] Deng L., Yu D. (2014). Deep Learning: Methods and Applications. Found. Trends Signal Process..

