# Supplementary material for: Electroencephalography-Based Brain–Machine Interfaces in Older Adults: A Literature Review
Source: Bioengineering (Basel). 2023 Mar 23;10(4):395. doi: 10.3390/bioengineering10040395 (PMC10136126; doi:10.3390/bioengineering10040395)
Supplement: Supplementary file 1 [file bioengineering-10-00395-s001.zip › bioengineering-2218953-supplementary.pdf]

bioengineering

Systematic Review

Electroencephalography-Based Brain–Machine Interfaces in Older Adults: A Literature Review

Luca Mesin [1,*], Giuseppina Elena Cipriani [2] and Martina Amanzio [2]

1 Mathematical Biology and Physiology, Department Electronics and Telecommunications, Politecnico di Torino, 10129 Turin, Italy
2 Department of Psychology, Universitá di Torino, 10124 Torin, Italy
* Correspondence: luca.mesin@polito.it; Tel.: +39-0110-904-085

Supplementary material

References of the excluded studies and reasons for the exclusion are listed in Table S1. A summary of the results and perspectives of the selected papers is shown in Table S2

Citation: Mesin, L.; Cipriani, G.E.; Amanzio, M. Electroencephalography- Based Brain–Machine Interfaces in Older Adults: A Literature Review. *Bioengineering* **2022**, *10*, 395. https://doi.org/10.3390/bioengineering10040395

Academic Editor: Mark L.Weiss

Received: 30 January 2023
Accepted: 21 March 2023
Published: 23 March 2023

Publisher's Note: MDPI stays neutral with regard to jurisdictional claims in published maps and institutional affiliations.

Copyright: © 2022 by the authors. Submitted for possible open access publication under the terms and conditions of the Creative Commons Attribution (CC BY) license (https://creativecommons.org/licenses/by/4.0/).

Table S1. Excluded studies and reasons for the exclusion.

Reference and Reason for Exclusion	
Schnakers C et al. Front Hum Neurosci. 2022;16:971315	not original article
Engemann DA et al. Neuroimage. 2022;262:119521	inadequate topic
Liu B et al. Sci Data. 2022;9(1):252	inadequate topic
Belkacem AN et al. Front Hum Neurosci. 2022;16:881922	not original article
Massetti N et al. J Alzheimers Dis. 2022;85(4):1639-1655	wrong procedure
Sponheim C et al. J Neural Eng. 2021;18(6)	wrong age group
Rahman MM et al. Comput Biol Med. 2021;136:104696.	not original article
Di Marco R et al. Methods Protoc. 2021;4(3):48	wrong age group
Schmitz S. Front Sociol. 2021;6:651486	not original article
Smith R et al. Sci Rep. 2021;11(1):10128.	wrong age group
Pavlov AN et al. Sensors (Basel). 2020;20(20):5843.	not original article
Belkacem AN et al. Front Neurosci. 2020;14:692	not original article
Marquez-Chin C et al. Biomed Eng Online. 2020;19(1):34	not original article
Cell. 2020;181(1):22-23	wrong age group
Welle EJ et al. J Neural Eng. 2020;17(2):026037	inadequate topic
Ali JI et al. J Int Neuropsychol Soc. 2020;26(1):31-46	not original article
Tang W et al. Artif Intell Med. 2020;102:101755.	wrong age group
Sepúlveda P et al. Anaesthesia. 2020;75(2):196-201	wrong age group
Shim S et al. Biomed Mater Eng. 2020;30(5-6):497-507	wrong species
Kang YN et al. IEEE Trans Neural Syst Rehabil Eng. 2019;27(6):1312-1319	inadequate topic
Martins NRB et al. Front Neurosci. 2019; 13:112	wrong age group
Beveridge R et al. IEEE Trans Neural Syst Rehabil Eng. 2019;27(4):572-581	wrong age group
Classen S et al. 2019;39(2):97-107	not original article
Woods V et al. J Neural Eng. 2018;15(6):066024	wrong species
Luo J et al. J Neural Eng. 2018;15(5):056015	wrong age group
Norton JJS et al. J Neural Eng. 2018;15(5):056012	wrong age group
Semprini M et al. Front Neurol. 2018;9:21	not original article
Brockmann PE et al. Eur J Paediatr Neurol. 2018;22(3):434-439	wrong age group
Fu TM et al. Proc Natl Acad Sci U S A. 2017;114(47):E10046-E10055	wrong species
Jiang Y et al. Front Aging Neurosci. 2017;9:52	not original article
Rana M et al. Front Aging Neurosci. 2016;8:239	not original article
Young KL et al. Accid Anal Prev. 2017;106:460-467	not original article
Kober SE et al. Neurobiol Aging. 2016;40:127-137	wrong age group
Wagner J et al. J Neurosci. 2016;36(7):2212-26	wrong age group
Murphy MD et al. Front Cell Neurosci. 2016;9:497	not original article
Hsu HT et al. IEEE Trans Neural Syst Rehabil Eng. 2016;24(5):603-15	wrong age group
Reichert JL et al. Int J Psychophysiol. 2016;99:67-78	inadequate topic
Goodman G et al. J Integr Neurosci. 2015;14(3):281-93	wrong age group
Tseng KC et al. Sensors (Basel). 2015;15(3):5518-30	wrong age group
Reichert JL et al. Clin Neurophysiol. 2015;126(11):2068-77	wrong age group

McCane LM et al. Clin Neurophysiol. 2015;126(11):2124-31	wrong age group
Gomez-Pilar J et al. Annu Int Conf IEEE Eng Med Biol Soc. 2014;2014:3630-3	not original article
Nuyujukian P et al. J Neural Eng. 2014;11(6):066003	wrong species
Young BM et al. Expert Rev Med Devices. 2014;11(6):537-9	not original article
Di Pino G et al. Front Syst Neurosci. 2014;8:109	not original article
Mandal HS et al. Acta Biomater. 2014;10(6):2446-54	inadequate topic
Ninaus M et al. Front Hum Neurosci. 2013;7:914	wrong age group
Guggenmos DJ et al. Proc Natl Acad Sci USA. 2013;110(52):21177-82	wrong age group
Aloise F et al. Clin EEG Neurosci. 2011;42(4):219-24	wrong age group
Li Y et al. PLoS One. 2011;6(6):e20801	wrong age group
Contreras-Vidal JL et al. Annu Int Conf IEEE Eng Med Biol Soc. 2010;2010:2825-8	not original article
Allison B et al. IEEE Trans Neural Syst Rehabil Eng. 2010;18(2):107-16	wrong age group
Lin CT et al. Gerontology. 2010;56(1):112-9	not original article

Table S2. Results and perspectives of the papers fitting our selection criteria.

Reference	Results	Perspectives
Li et al., 2022 [7]	Older adults are less affected by the degree of cognitive fatigue during MI, compared to young participants.<br><br>Nevertheless, MI energy is lower in the older population, than in younger people.	BCI-MI in the older population needs not to be based on SMR alone and the appropriate algorithms can be applied without obvious lateralization of ERD.<br><br>CNN model based on fused spatial information greatly improves classification accuracy and leads to longer training time.
Goelz et al., 2021 [8]	The results confirm an age-related reorganization of brain networks and show a correlation with task characteristics. Electrophysiological brain activity patterns associated with altered sensorimotor network in older adults, suggesting<br>reorganization of task-related brain networks.	Future research on BCI applications should consider age-related differences in the development of BCI and NF systems when targeting the older population<br>(e.g., in the selection of appropriate features and algorithms).
Chen et al., 2019 [9]	Decreased cortical lateralization of the somatosensory cortex and overall reduction in EEG power in older subjects. This resulted in lower accuracy of BCI classification based on spatial activation information. Older subjects showed less lateralization in somatosensory cortex in response to vibro-tactile stimulation compared to younger adults.	Future studies should focus on the effects of ageing on EEG signals.<br><br>In addition, NFT methods to improve cortical lateralization and algorithms not based solely on EEG lateralization should be investigated. These age-related EEG changes reflected greater susceptibility to noise and interference.
Zich et al., 2017 [10]	Brain activity patterns show lower lateralization of ERD % and HbR concentration during MI, but not ME, in older subjects compared with younger participants.	Age-related changes in MI should be taken into account when designing MI NF protocols for patients. The influence of age must be considered in the design of neuro-rehabilitation protocols for stroke patients. Complex relationship between age and exercise-related activity in both EEG and hemodynamic measurements.
Herweg et al, 2016 [11]	Tactile BCI performance is valuable although age-related changes in somatosensory abilities are negligible. The protocol enabled learning and significantly improved BCI performance and EEG characteristics, demonstrating the positive effect of training.	Future studies should focus on tactile BCI development, considering specific stimulation design, individual characteristics and training. Tactile BCIs can be a valid alternative to visual and auditory tasks. They can<br>be used despite age-related changes in somatosensory abilities.
Gomez-Pilar et al., 2016 [12]	Usefulness of NF training with a motor imagery-based BCI in terms of improvements in all cognitive functions except attention.	This study may be helpful in the development of new NF training based on MI strategies, useful in rehabilitating cognitive functions<br>by improving brain plasticity and neuropsychological functions, which seems to affect the older population.
Karch et al., 2015 [13]	WM load and spatial attentional focus could be distinguished in all age comparison groups based on EEG responses in alpha range.	A multivariate approach provides better discrimination than classical non-person-specific models,<br>at both the individual and group levels.
Lee et al., 2015 [14]	Improvements in attention and delayed memory before and after CT. No significant changes observed<br>in immediate memory and visuospatial/constructive areas.	BCI-based intervention showed promising results<br><br>in improving memory and attention. This intervention could potentially reduce or prevent cognitive decline

		in patients with mild or major neurocognitive disorders.
Lee et al., 2013 [15]	Significant improvements in immediate memory, visuospatial/constructive, attention and delayed memory before and after CT.	BCI-based intervention showed promising results in improving memory and attention. This intervention could potentially reduce or prevent cognitive decline in patients with mild or major neurocognitive disorders.
